# Estimation of feasible solution space using Cluster Newton Method: application to pharmacokinetic analysis of irinotecan with physiologically-based pharmacokinetic models

**DOI:** 10.1186/1752-0509-7-S3-S3

**Published:** 2013-10-16

**Authors:** Kenta Yoshida, Kazuya Maeda, Hiroyuki Kusuhara, Akihiko Konagaya

**Affiliations:** 1 Laboratory of Molecular Pharmacokinetics, Graduate School of Pharmaceutical Sciences, The University of Tokyo, Tokyo, Japan; 2 Department of Computational Intelligence and Systems Science, Tokyo Institute of Technology, Yokohama, Japan

**Keywords:** Pharmacokinetics, PBPK models, Optimization

## Abstract

**Background:**

To facilitate new drug development, physiologically-based pharmacokinetic (PBPK) modeling methods receive growing attention as a tool to fully understand and predict complex pharmacokinetic phenomena. As the number of parameters to reproduce physiological functions tend to be large in PBPK models, efficient parameter estimation methods are essential. We have successfully applied a recently developed algorithm to estimate a feasible solution space, called Cluster Newton Method (CNM), to reveal the cause of irinotecan pharmacokinetic alterations in two cancer patient groups.

**Results:**

After improvements in the original CNM algorithm to maintain parameter diversities, a feasible solution space was successfully estimated for 55 or 56 parameters in the irinotecan PBPK model, within ten iterations, 3000 virtual samples, and in 15 minutes (Intel Xeon E5-1620 3.60GHz × 1 or Intel Core i7-870 2.93GHz × 1). Control parameters or parameter correlations were clarified after the parameter estimation processes. Possible causes in the irinotecan pharmacokinetic alterations were suggested, but they were not conclusive.

**Conclusions:**

Application of CNM achieved a feasible solution space by solving inverse problems of a system containing ordinary differential equations (ODEs). This method may give us reliable insights into other complicated phenomena, which have a large number of parameters to estimate, under limited information. It is also helpful to design prospective studies for further investigation of phenomena of interest.

## Background

Pharmacokinetics is a field of study that analyzes and predicts behaviors of drugs in organisms [1]. A major purpose of this study area is to predict pharmacokinetic properties of new drugs in humans, without performing clinical studies, in order to accelerate the efficiencies of new drug development processes. Another important purpose is to facilitate the proper use of not only newly developed drugs but also already existing drugs. There are numerous factors altering pharmacokinetics, such as drug-drug interactions (DDIs) [1], pharmacogenetics [2], or disease states [3,4], which can cause large inter-individual variability in drug responses. By studying these complicated phenomena, we may be able to explain and predict the alterations in clinical settings to administer drugs properly to each patient [5].

Physiologically-based pharmacokinetic (PBPK) modeling and simulation are essential in understanding and predicting the above-mentioned, complicated pharmacokinetic phenomena [6-8]. The basic idea of PBPK modeling is to reproduce physiological functions (absorption, distribution, metabolism, and elimination) in mathematical equations in order to understand the physiological phenomena extensively from the information obtained from *in vivo *studies and to predict unknown phenomena quantitatively. PBPK models are becoming more important because of the increase in complicated pharmacokinetic phenomena, such as DDIs involving multiple interaction sites [9] or the combined effect of multiple inhibitors [10]. Current draft guidance on DDI studies by the U.S. Food and Drug Administration [11] emphasizes the importance of PBPK simulation in deciding whether clinical DDI studies are required or not during new drug developments.

In analyzing complex pharmacokinetic phenomena with PBPK models, Gauss-Newton or its modified algorithm is often used for parameter estimation. However, these methods require feasible initial parameters beforehand, which are often difficult for the pathways with little or no prior information, such as pharmacokinetic parameters of metabolites, or of enterohepatic circulations (EHC). Optimized parameters may highly depend on the initial parameter settings as well. Additionally, the PBPK model contains a lot of parameters to estimate in nature, compared to the limited information available in clinical studies, making the accurate estimation of parameters more difficult. Since the accuracy of the parameter estimation process is quite important in PBPK analyses, these characteristics make the interpretations or extrapolations of the obtained results complicated.

Aoki et al. recently developed a new parameter estimation algorithm called CNM [12]. In this algorithm, we first prepare a group of virtual samples with random samplings from a certain initial range for each parameter to estimate. Then, linear approximations of a projection from parameter space into target values give the initial parameter values for the next iteration. In our experience, fewer than nine iterations of this process achieve the final, optimized parameters, which can reproduce clinically observed phenomena.

This algorithm has multiple advantages over conventional parameter estimating algorithms. The first advantage is the simplicity of the initial parameter settings. The new method only requires the designation of relatively broad parameter ranges as an initial setting, while the conventional algorithm requires the identification of feasible initial parameters. The second advantage is low computational costs owing to the deterministic nature of this algorithm, unlike other algorithms that use collective intelligence, such as genetic algorithms or particle swarm optimization. The last and the most important advantage is that the results are obtained as a group of optimized parameter sets. It allows us to interpret the phenomena with higher confidence and to extrapolate the obtained insights into new phenomena.

**Figure 1:**
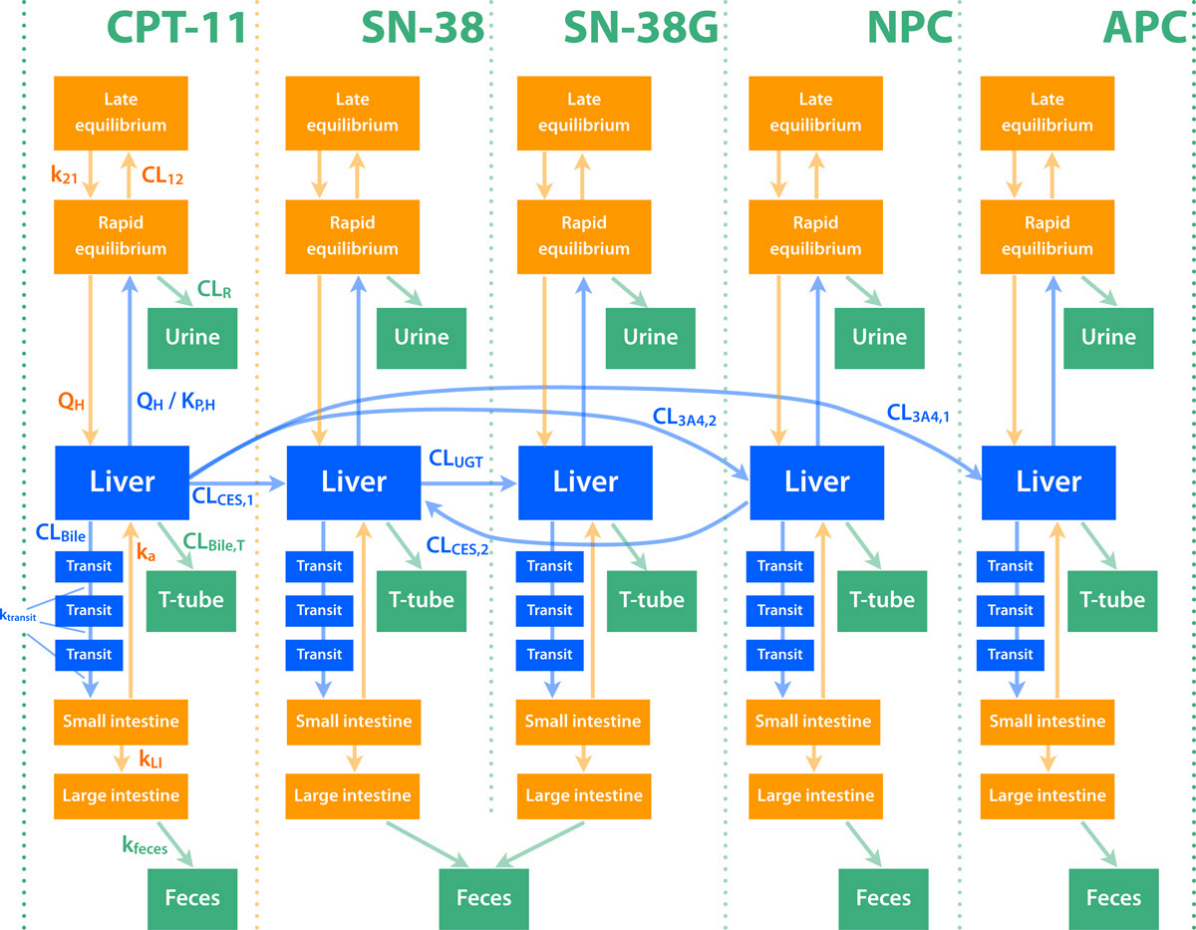
**A PBPK model to analyze pharmacokinetic properties of irinotecan and metabolites**. We constructed a PBPK model to simulate time profiles of the accumulations of irinotecan and the metabolites. We included compartments for rapid and late equilibrium, liver, small and large intestine, biliary transits, urine, feces, and biliary T-tubes.The ordinary differential equations of this model are described in the Additional file 1. CL_12_, clearance from a rapid to a late equilibrium compartments; CL_CES,1_, metabolic clearance of irinotecan by CES2 to form SN-38; CL_CES,2_, metabolic clearance of NPC by CES2 to form SN-38; CL_bile_, biliary clearance to a transit compartment; CL_bile,T_, biliary clearance to biliary T-tube; CL_3A4,1_, metabolic clearance of irinotecan by CYP3A4 to form APC; CL_3A4,2_, metabolic clearance of irinotecan by CYP3A4 to form NPC; CL_R_, renal clearance; CL_UGT_, metabolic clearance of SN-38 by UGT to form SN-38G; CPT-11, irinotecan; k_21_, kinetic constant from a late to a rapid equilibrium compartment; k_a_, absorption rate constant; k_feces_, kinetic constant for the transit from large intestine to feces; k_LI_, kinetic constants for the transit from small intestine to large intestine; K_P,H_, concentration ratio between liver and rapid equilibrium compartment; k_transit_, kinetic constant for the transit in bile compartments to small intestine; Q_H_, blood flow rate in liver.

Aoki et al. previously applied CNM for analyzing a complicated pharmacokinetic phenomenon [5,12] using a PBPK model [13], in which the pharmacokinetic properties of an anti-cancer drug, irinotecan (also known as CPT-11), differs a lot between a bile-duct cancer (BDC) patient and other cancer (OC) patients [14]. After intravenous administration, irinotecan is metabolized by CYP3A4 or carboxylesterase 2 (CES2) to form APC, NPC, SN-38, and M4 [15,16] (Figure 1). NPC and SN-38 are further metabolized by CES2 and UGT1A to form SN-38 and SN-38G, respectively. Organic anion transporting polypeptide 1B1 (OATP1B1) is involved in the hepatic uptake of SN-38, while OATP1B1 does not actively transport irinotecan or SN-38G, according to an *in vitro *study [17]. SN-38G is said to be deconjugated to form SN-38 by β-glucuronidase in intestinal microflora [18]. Possible involvement of enterohepatic circulation (EHC) in determining irinotecan pharmacokinetics [19] makes the estimation of parameters difficult because of the model structure and limited information about the feasible parameter values of EHC.

Since the previous report is mainly focused on the establishment of CNM, the PBPK model was not suitable from the pharmacokinetic viewpoint. Most importantly, the previous PBPK model did not contain EHC. Biliary drainage in the BDC group was not considered either. Furthermore, our preliminary investigation showed that the original CNM is not applicable to analyze the phenomena with EHC in a PBPK model.

In this paper, we improved both the PBPK model of irinotecan and the CNM algorithm itself. Firstly, we included EHC and biliary drainage in the PBPK model to properly interpret our obtained results. We also tried to improve the CNM algorithm itself to maintain the diversity of virtual samples during iterations, since the application of the original CNM algorithm failed using the new PBPK model in this study.

## Results and discussion

### Improvements in CNM for analyzing irinotecan accumulation profiles

In this study, we have applied and improved the CNM algorithm for the accumulation profiles of irinotecan and its metabolites by using the PBPK model shown in Figure 1 and in Additional File 1. Parameter estimation in the PBPK model was performed with 9 and 14 objective values for OC and BDC, respectively (Table 1c). Initial ranges of parameters were set as shown in Table 1b, which were thought to be large enough to contain the real value for each parameter.

**Table 1:** Initial, final, and fixed parameters for CNM

**a **Parameters Fixed				
	
	Dose	µg/kg	1500	
	
	Duration of infusion	Min	90	
	
	Q_liver_	ml/min/kg	20.7	
	
	V_liver_	ml/kg	24.1	
				
**b **Parameters to estimate				
ID			min	Max
1-5	K_p,liver_	-	0.1	10

6-11	CL_r_	ml/min/kg	1	100

11-15	CL_bile_	ml/min/kg	1	100

16	CL_CES,1_	ml/min/kg	1	100

17	CL_CES,2_	ml/min/kg	1	100

18	CL_3A4,1_	ml/min/kg	1	100

19	CL_3A4,2_	ml/min/kg	1	100

20	CL_UGT_	ml/min/kg	1	100

21-25	k_feces_	/min/kg	0.001	0.1

26-30	k_a_	/min/kg	0.001	0.1

31-35	k_LI_	/min/kg	0.001	0.1

36-40	k_transit_	/min/kg	0.001	0.1

41-45	CL_12_	ml/min	1	100

46-50	k_21_	min^-1^	0.1	10

51-55	V_rapid_	ml/kg	10	1000

56*	CL_bile,T-tube_/CL_bile,transit_	-	0.1	10

We used the same initial ranges for these parameters in irinotecan and the metabolites				
* Fixed and set to be zero when analyzing the OC group.				
				
**c **Objective values				
ID			OC	BDC

1	Urinary accumulation µg/kg	irinotecan	427	412
		
2		SN-38	8.20	17.0
		
3		SN-38G	57.6	227
		
4		NPC	2.67	1.70
		
5		APC	42.5	146

6	Fecal accumulation µg/kg	irinotecan	616	118
		
7		SN-38 + SN-38G	162	52.6
		
8		NPC	25.9	6.05
		
9		APC	158	31.4

10	Biliary accumulation µg/kg	irinotecan	-	349
		
11		SN-38	-	8.32
		
12		SN-38G	-	50.5
		
13		NPC	-	5.68
		
14		APC	-	73.8

**a **fixed parameters for calculation, **b **initial parameters to estimate, and **c **objective values are shown. Minimum and maximum values of the initial ranges were represented for the parameters to estimate. We used the same initial ranges for each of the five parameters for irinotecan and the metabolites, except for #16-20 and #56. We used the same parameter value of #56 for irinotecan and the metabolites. Parameter #56 was fixed and set to be zero and fixed when analyzing the OC group. The total amount of urinary, fecal, and biliary accumulation was normalized to the amount of dose (1500 µg/kg). BDC, bile-duct cancer patients; CL_12_, clearance from a rapid to a late equilibrium compartments; CL_CES,1_, metabolic clearance of irinotecan by CES2 to form SN-38; CL_CES,2_, metabolic clearance of NPC by CES2 to form SN-38; CL_bile,total_, sum of biliary clearance to a transit compartment and biliary clearance to biliary T-tube; CL_3A4,1_, metabolic clearance of irinotecan by CYP3A4 to form APC; CL_3A4,2_, metabolic clearance of irinotecan by CYP3A4 to form NPC; CL_R_, renal clearance; CL_UGT_, metabolic clearance of SN-38 by UGT to form SN-38G; k_21_, kinetic constant from a late to a rapid equilibrium compartment; k_a_, absorption rate constant; k_feces_, kinetic constant for the transit from large intestine to feces; k_LI_, kinetic constants for the transit from small intestine to large intestine; K_P,H_, concentration ratio between liver and rapid equilibrium compartment; k_transit_, kinetic constant for the transit in bile compartments to small intestine; OC, other cancer patients; Q_H_, blood flow rate in liver; V_H_, volume of a liver; V_rapid_, apparent volume of a rapid equilibrium compartment.

**Figure 2:**
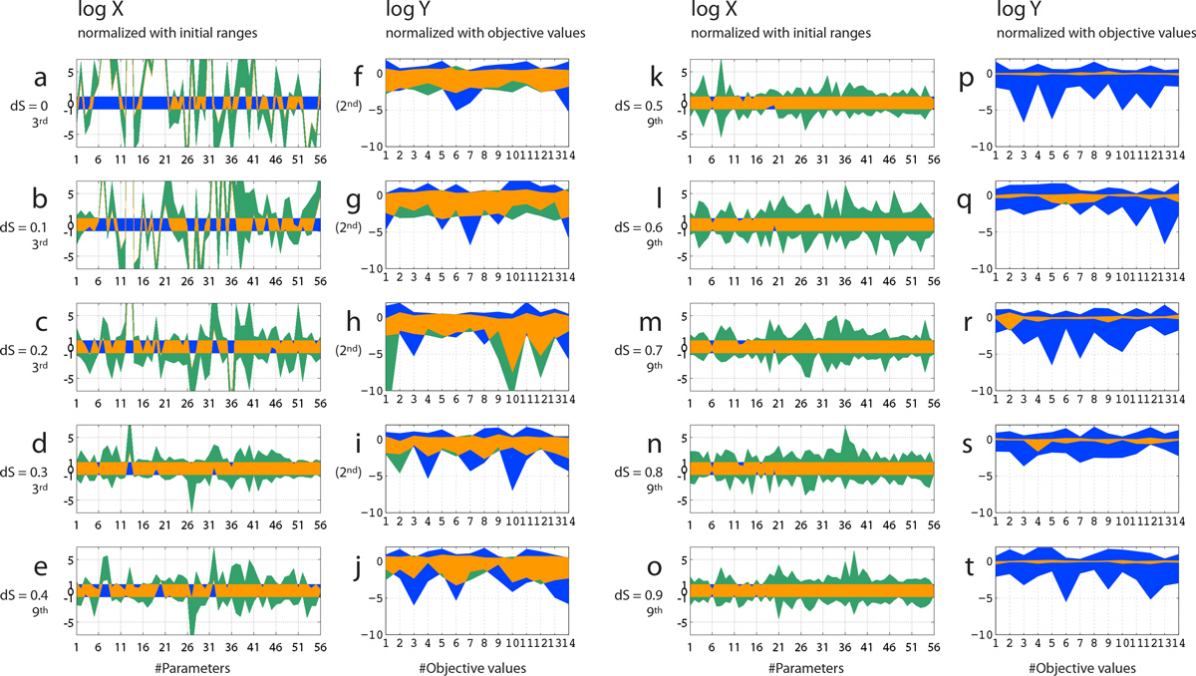
**Effect of dS values on convergences of parameters and objective values in the BDC group**. Distributions of parameters to estimate and objective values at the final iterations in BDC group with corresponding dS values are displayed for the initial (blue) and estimated (green) ranges. Overlapped areas are represented in orange. A number of iterations and a dS value are displayed at the lefthand side of each figure. Parameters got diverged after 2 iterations for dS of 0 to 0.2, or after 3 iterations for dS of 0.3. Normalized values with the initial ranges in log-scale are displayed for the parameters to estimate, and normalized values with the observed objective values in log-scale are displayed for the simulated objective valued. BDC, bile-duct cancer patients.

Firstly, we performed the modified CNM with various dS values, ranging from 0 to 0.9. The modified CNM replicates a group of parameters towards the direction of the estimated feasible solution space with the ratio of dS after each iteration (see **Methods **section for details). We examined the effect of dS values on the convergence in this system, which was newly introduced in the algorithm to maintain parameter diversities as explained in the **Methods **section, and the calculation with dS of 0 is identical to the original CNM algorithm. By changing the dS values from 0 to 0.9, we performed CNM on the accumulation profiles in the BDC group with the same initial parameter ranges. As shown in Figure 2, the objective values converged well to the observed values when dS was no less than 0.5 (Figure 2p-t). For the OC group, dS of at least 0.2 was needed to complete the process (data not shown).

When dS was smaller than 0.5, the ranges of the objective values remained similar to the initial range (Figure 2f-j). Particularly, parameters got diverged after 2 iterations for dS of 0 to 0.2, or after 3 iterations for dS of 0.3 (Figure 2a-d). The divergences of parameters after 3 iterations were smaller with higher dS values. It might be due to the capability to maintain parameter diversities with higher dS values. Since we have some parameters sensitive to the changes in dS values, such as a biliary clearance to a transit compartment of SN-38 (CL_bile_, parameter #12), we might be able to control the convergence by observing the behaviors of sensitive parameters. These results suggest the importance of maintaining parameter diversities in performing CNM using the dS algorithm, while further investigations should be needed to rationally determine feasible dS values in other cases.

**Figure 3:**
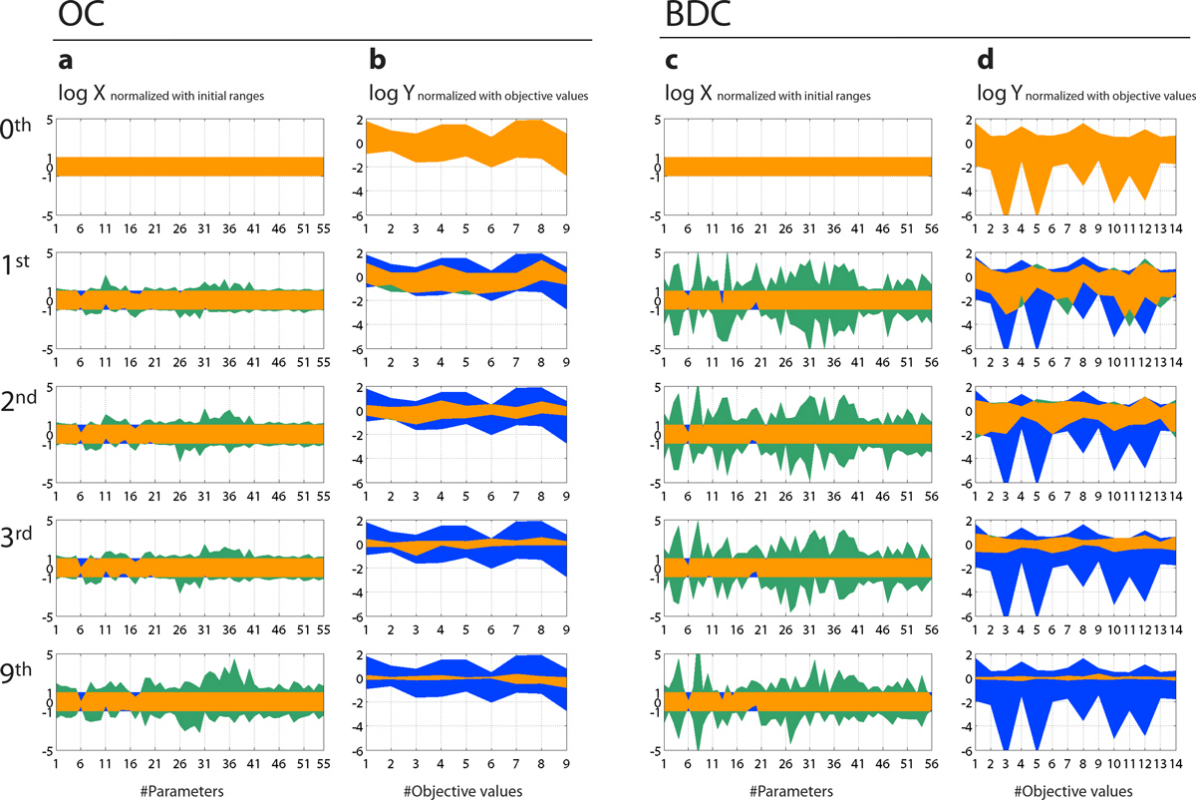
**Normalized fitted parameters and target values over iterations**. Processes of parameter estimations for (a-b) OC and (c-d) BDC groups are described for the initial (blue) and estimated (green) ranges with dS of 0.5. Overlapped areas are represented in orange. Convergence of (a,c) parameters and (b,d) objective values can be observed. Normalized values with the initial ranges in log-scale were displayed for the parameters to estimate (a,c), and normalized values with the observed objective values in log-scale were displayed for the simulated objective valued (b,d).Corresponding parameter ranges after nine iteractions were described in the Additional File 2. BDC, bile-duct cancer patients; OC, other cancer patients.

Some pharmacokinetic parameters gradually converged to a feasible solution space along with the iterations of the parameter estimation process with high dS values. The residuals in objective values were decreased towards zero as well. Figure 3 illustrates the estimated parameters and the objective values after iterations with dS of 0.5, and the corresponding values are described in Additional File 2. While the ranges of parameter values seem stable only after the first iteration, the residuals in objective values continued to decrease until the third iterations. This observation may suggest that correlations among parameter values converged while maintaining the diversity of the absolute value in each parameter.

After 9 iterations, we observed high convergence of certain parameters, such as renal clearance (CL_R_) of irinotecan (parameter #6) in both the OC and the BDC groups, and ratio of biliary clearance into T-tube to biliary clearance into a transit compartment (CL_bile,T _/ CL_bile,transit_, parameter #56) in the BDC group (differences in convergence will be discussed later). In particular, the estimated values of irinotecan CL_R _in OC (5.7 ± 1.2 ml/min/kg, geometric mean ± SD, Additional file 2) was close to the values calculated from the original report (2.8 ml/min/kg). Furthermore, the averages of estimated irinotecan CL_R _values with two different initial distributions were within three fold of the values from the original report (data not shown). We do not have clear explanations of the convergences in clearance parameters without information on plasma concentrations, since these parameters are defined as the ratio between urinary, biliary, or fecal accumulations of drugs with area under the blood concentration-time curve (AUC). We suspect that these parameters were determinants of the behavior of our PBPK model structures, and that the relationships with other parameters restricted the absolute values.

The calculation time required for the whole process was short; it took less than 15 minutes with ten iterations and 3000 virtual samples (Intel Xeon E5-1620 3.60GHz × 1 or Intel Core i7-8702.93GHz × 1; see **Methods **section for details).

**Figure 4:**
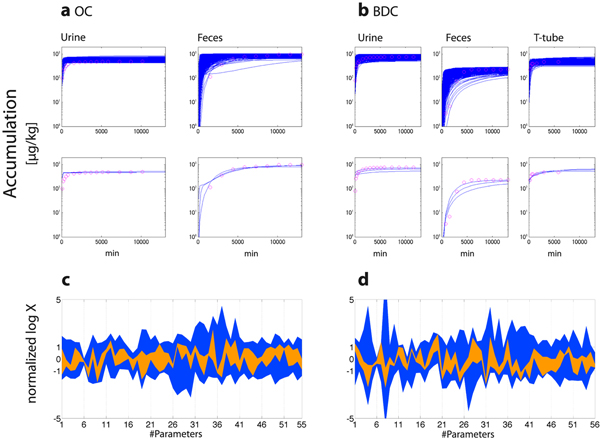
**Simulated accumulation time-profiles after parameter estimations and the parameter selections with SS_log_**. Accumulation time-profiles of total irinotecan radioactivity in urine, feces, and biliary T-tubes were simulated using the estimated parameters with CNM for (a) OC and (b) BDC, after the estimation processes with the objective values shown in Table 1c. Blue lines in the upper and lower panels represent the simulated time-profiles with all the estimated parameter sets and the parameter sets with three lowest SS_log _values. Pink circles represent the observed time-profiles. Distributions of parameters to estimate at the final iterations were displayed for all the parameter sets (blue) and the parameter sets with three lowest SS_log _values (green) for (c) OC and (d) BDC. Overlapped areas are represented in orange. Normalized values with the initial ranges in log-scale are displayed for the parameters to estimate. BDC, bile-duct cancer patients; OC, other cancer patients; SS_log_, sum of squares of log residuals.

After the application of CNM, we simulated the accumulation time-profiles using estimated parameter sets to compare them with the reported values (Figure 4a, b). In both groups, some parameter sets reproduced the observed time profiles well, and the range of the estimated parameters became smaller when we selected samples which reproduced the observed time profile well (Figure 4c, d).

### Differences in optimized parameters between OC and BDC

**Figure 5:**
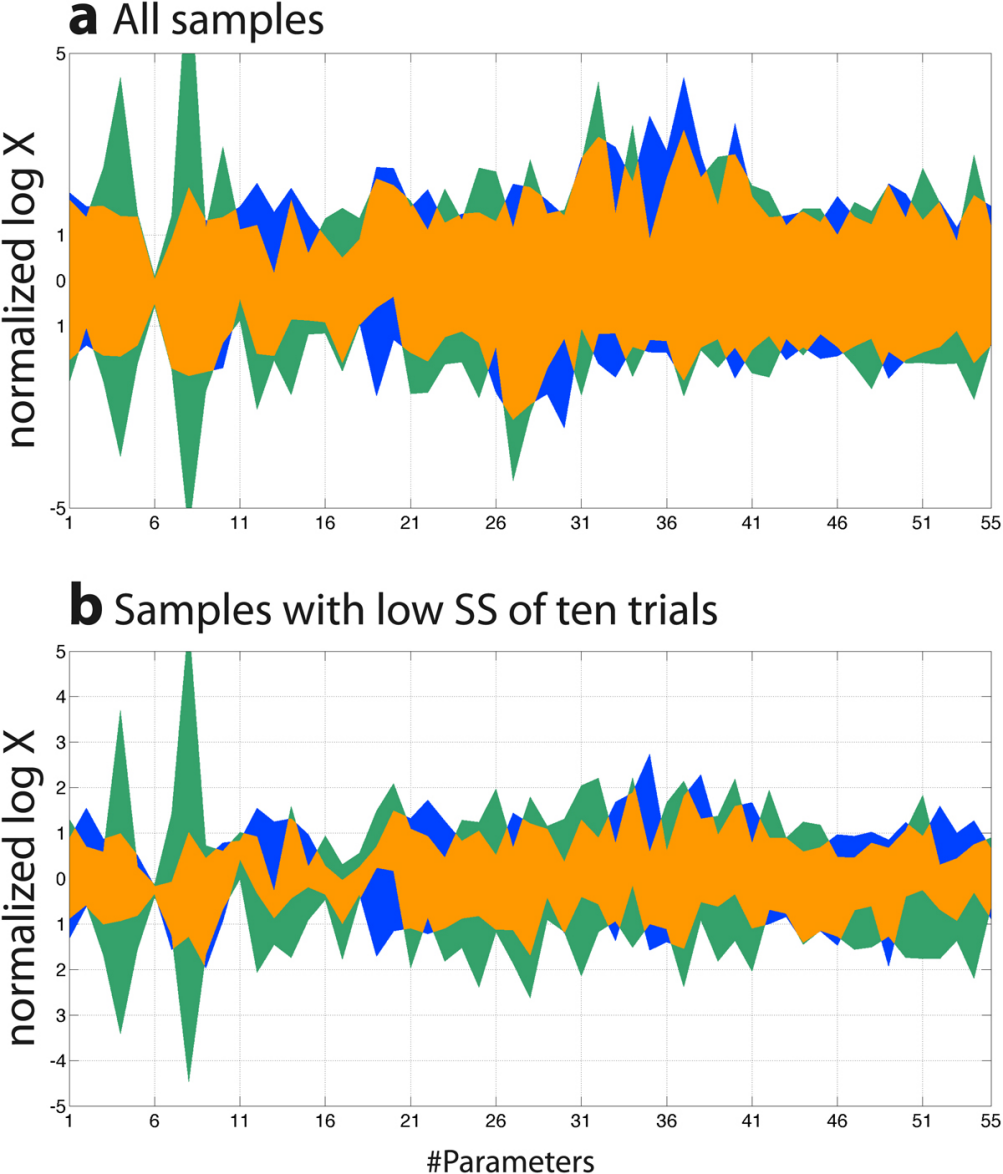
**Comparison of estimated parameters for OC and BDT**. Estimated parameter distributions were compared between OC (blue) and BDT (green) for (a) all the estimated parameter sets and (b) the parameter sets with ten lowest SS_log _values. Overlapped areas are represented in orange. Normalized values with the initial ranges in log-scale are displayed for the parameters to estimate. BDC, bile-duct cancer patients; OC, other cancer patients; SS_log_, sum of squares of log residuals.

We compared the estimated parameter distributions between OC and BDC. When comparing the distribution of parameters with all samples, parameter distributions were similar in the two groups (Figure 5a). As mentioned in the previous section, we observed strong convergence in parameter #6, CL_R _of irinotecan. Expansions of parameter ranges were also similar between the two groups. On the other hand, the distributions of the numbers of parameters were shifted when we considered accumulation time-profiles (Figure 5b). Interestingly, these parameters are mostly involved in hepatobiliary or intestinal pathways (#12-13, 19, 20, 35, 41). These results may suggest the importance of hepatobiliary and intestinal pharmacokinetic processes in determining the pharmacokinetics of irinotecan in the BDC patient, and are partly in a good agreement with the reports on reduced MRP2 expressions in liver with biliary cancer [20] or in the duodenums of patients with hepatic cholestasis [21], and altered pharmacokinetics of irinotecan and its metabolites with genetic polymorphisms of MRP2 [15,16]. However, further investigations are needed to clarify the cause of the pharmacokinetic alterations, since most of the parameter ranges in BDC group are actually included in the parameter ranges in OC group.

### Correlations among parameters

**Figure 6:**
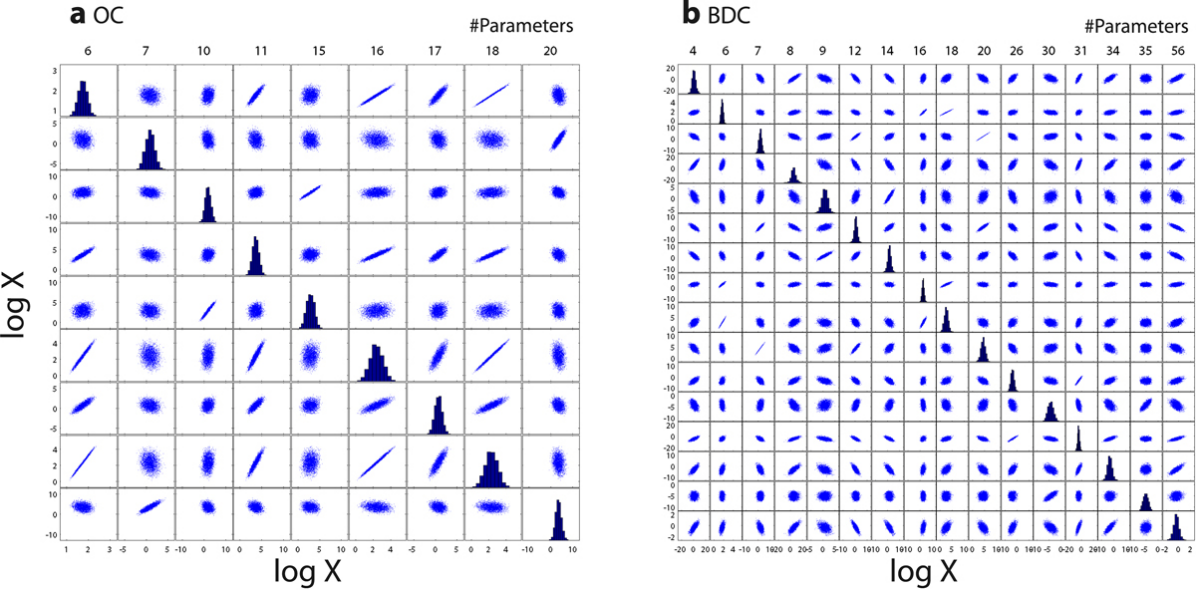
**Correlations among estimated parameters**. Correlations of parameters (a) OC and (b) BDC groups were visualized using PLOTMATRIX function in MATLAB. Parameters to be displayed were those who have high correlation (r^2^> 0.64) with at least one other parameter. BDC, bile-duct cancer patients; OC, other cancer patients; SS_log_, sum of squares of log residuals.

Finally, we have observed correlations among the estimated parameter values (Figure 6). We selected parameters with an *r^2 ^*value of more than 0.64 against at least one other parameter in this figure. Tendencies in parameter correlations were similar between the two groups, while the number of parameter combinations having higher correlations was large in BDC group. The number of objective values might again be the cause of this observation.

Most of these relationships can be explained theoretically. For example, in both groups, parameters among #6, 16, and 18 have a strong linear relationship with each other to keep the ratio of parameters to maintain the ratio in the final outcome. Another example is the inverse proportional relationship of #56 to #12 and #14. When the amount of bile collected in the T-tube was increased (#56 increased), biliary clearances decreased (#12 and #14) to keep the amount of compounds excreted into the T-tubes. While these phenomena can be explained retrospectively, we cannot easily observe the actual parameter dependencies quantitatively without performing this kind of parameter estimation algorithms.

## Conclusions

Efficient and reliable estimations of parameter values are important for the analysis of complex pharmacokinetic phenomena with PBPK models. In this study, we have successfully improved and applied CNM to estimate 55 or 56 parameters in the irinotecan PBPK model, by implementing dS function in the algorithm. The application of this method presented not only the control parameters in this PBPK model, but also correlations of parameters, which are important in determining the behaviors of the PBPK model.

## Methods

### Data source

Throughout thee study, we analyzed reported pharmacokinetic profiles of irinotecan and its metabolites [14]. This report described the accumulation of irinotecan and its metabolites into urine and feces for two groups of patients, with bile-duct cancer (BDC, one patient) or with other cancers (OC, seven patient). Bile was also collected via biliary T-tube for a patient in the BDC group.

### PBPK model and parameter settings

In order to analyze the causes of the different pharmacokinetic profiles from the two groups of patients, we developed a simplified PBPK model for irinotecan and its four metabolites (Figure 1), based on the reported metabolic pathways of irinotecan [15,16]. The ordinary differential equations of this model are described in the Additional File 1.

Fixed parameter values and initial parameter ranges were shown in Table 1a and 1b, respectively. While these ranges were set arbitrarily, they may be large enough to contain real values, and may be applicable to many compounds having different kinetic constants. We used the same parameter for the ratio between CL_bile,T _and CL_bile _for irinotecan and the metabolites, since this parameter can be regarded as the ratio of bile flow between in T-tube and in bile duct after T-tube. In OC group, CL_bile,T- _was set to be equal to zero, since biliary T-tube was not placed for this group of patients. We have performed the whole parameter optimization processes in the log space of all the parameters for avoiding negative parameter values in the original space.

Objective values for the parameter estimation processes were shown in Table 1c. Since the deconjugation of SN-38G to SN-38 by β-glucuronidase in intestinal microflora may affect the fecal elimination amount of SN-38G and SN-38, we combined the accumulation of these two compounds in objective values #7.

### Materials

We solved the system of ODEs by using the MATLAB stiff ODE solver ODE15s [22]. We performed all calculations with MATLAB using a desktop computer (CPU: Core i7-870 2.93GHz × 1, OS: Windows 7 SP1 32 bit, RAM: 4GB, MATLAB version: 8.0.0) or a workstation (CPU: XeonE5-1620 3.60GHz × 1, OS: CentOS 6.4 64 bit, RAM: 16GB, MATLAB version: 8.1.0).

### CNM Method and its modification

CNM was constructed previously [12]. Briefly, a group of initial parameter sets (3000 virtual samples) was prepared with a random sampling from given parameter ranges. The linear approximations of the projections from one group of parameter sets into objective values generated the next group, and nine iterations of this process yielded a group of optimized parameter sets.

In each iteration process, we have newly implemented a calculation using a parameter called dS to maintain parameter diversities. In each iteration of the original CNM, we have parameter sets before (X_b_) and after (X_a_) the parameter estimation process. In our new strategy, we calculated internally dividing point X_i _with the ratio of dS:(1-dS), and applied the same inverse matrix to obtain new estimated parameters X_a_'. Parameter sets for the next iteration were obtained by randomly selecting X_a _or X_a_' for each virtual sample.

After completing the estimations of parameters, the accumulation time-profiles were compared with the observed profiles, using sum of squares of log residuals (SS_log_):

$${SS}_{log}=\sum {\left(\text{ln}\frac{A_{e,simulated}}{A_{e,observed}}\right)}^{2}$$

where A_e,simulated _and A_e,observed _represent the simulated or observed amount of total irinotecan radioactivity at each time point.

### Correlations of parameters

After the parameter estimation process, we have observed parameter correlations using PLOTMATRIX function in MATLAB. Parameters to be displayed in Figure 6 were those who have high correlation (*r^2^*> 0.64) with at least one other parameter.

## List of abbreviations used

CL_12_, clearance from a rapid to a late equilibrium compartment; CL_CES,1_, metabolic clearance of irinotecan by CES2 to form SN-38; CL_CES,2_, metabolic clearance of NPC by CES2 to form SN-38; CL_bile,total_, sum of biliary clearance to a transit compartment and biliary clearance to biliary T-tube; CL_3A4,1_, metabolic clearance of irinotecan by CYP3A4 to form APC; CL_3A4,2_, metabolic clearance of irinotecan by CYP3A4 to form NPC; CL_R_, renal clearance; CL_UGT_, metabolic clearance of SN-38 by UGT to form SN-38G; CNM, Cluster Newton Method; CPT-11, irinotecan; k_21_, kinetic constant from a late to a rapid equilibrium compartment; k_a_, absorption rate constant; k_feces_, kinetic constant for the transit from large intestine to feces; k_LI_, kinetic constants for the transit from small intestine to large intestine; K_P,H_, concentration ratio between liver and rapid equilibrium compartment; k_transit_, kinetic constant for the transit in bile compartments to small intestine; Q_H_, blood flow rate in liver; V_H_, volume of a liver; V_rapid_, apparent volume of a rapid equilibrium compartment.

## Competing interests

The authors declare that they have no competing interests.

## Authors' contributions

K.Y. wrote the manuscript, designed research, performed research, and analyzed data. K.M. wrote the manuscript and designed research. H.K. wrote the manuscript and designed research. A.K. wrote the manuscript, designed research, and analyzed data. All authors read and approved the final manuscript.

## Supplementary Material

Additional file 1 The ordinary differential equations in our PBPK model Click here for file

Additional file 2 Estimated parameter ranges after nine iterations of CNM Click here for file

## Declarations

The publication costs for this article were funded by a Grant-in-Aid for Scientific Research on Innovative Area Molecular Robotics from the MEXT, Japan.

This article has been published as part of *BMC Systems Biology *Volume 7 Supplement 3, 2013: Twelfth International Conference on Bioinformatics (InCoB2013): Systems Biology. The full contents of the supplement are available online at http://www.biomedcentral.com/bmcsystbiol/supplements/7/S3.
