# Supplementary material for: Estimation of feasible solution space using Cluster Newton Method: application to pharmacokinetic analysis of irinotecan with physiologically-based pharmacokinetic models
Source: BMC Syst Biol. 2013 Oct 16;7(Suppl 3):S3. doi: 10.1186/1752-0509-7-S3-S3 (PMC3852065; doi:10.1186/1752-0509-7-S3-S3)
Supplement: Additional file 1 — The ordinary differential equations in our PBPK model [file 1752-0509-7-S3-S3-S1.pdf]

Additional file 1 – The ordinary differential equations in our PBPK model

The following ordinary differential equations were used throughout the study.

i in the equations corresponds to irinotecan or one of the metabolites; 1 – irinotecan, 2 – SN-38, 3 – SN-38G, 4 – NPC, 5 – APC.

- Rapid equilibrium compartments

$$V_{rapid,i} \cdot \dot{C}_{rapid,i} = k_{21,i} \cdot X_{late,i} + \frac{Q_H}{K_{P,H,i}} \cdot C_{liver,i} - (Q_H + CL_{12,i} + CL_{R,i}) \cdot C_{rapid,i}$$

- Late equilibrium compartments

$$\dot{X}_{late,i} = CL_{12,i} \cdot C_{rapid,i} - k_{21,i} \cdot X_{late,i}$$

- Small intestinal (SI) compartments

$$\dot{X}_{SI,i} = k_{transit,i} \cdot X_{transit3,i} - (k_{a,i} + k_{LI,i}) \cdot X_{SI,i}$$

- Large intestinal (SI) compartments

$$\dot{X}_{LI,i} = k_{LI,i} \cdot X_{SI,i} - k_{feces,i} \cdot X_{SI,i}$$

- Transit compartments

$$\dot{X}_{transit,1,i} = \frac{CL_{Bile,i}}{K_{P,H,i}} \cdot C_{liver,i} - k_{transit,i} \cdot X_{transit,1,i}$$
$$\dot{X}_{transit,2,i} = k_{transit,i} \cdot X_{transit,1,i} - k_{transit,i} \cdot X_{transit,2,i}$$
$$\dot{X}_{transit,3,i} = k_{transit,i} \cdot X_{transit,2,i} - k_{transit,i} \cdot X_{transit,3,i}$$

- Urine

$$\dot{X}_{Urine,i} = CL_{R,i} \cdot C_{rapid,i}$$

- Feces

$$\dot{X}_{feces,i} = k_{feces,i} \cdot X_{LI,i}$$

- Biliary T-tube

$$\dot{X}_{T-tube,i} = \frac{CL_{Bile,T,i}}{K_{P,H,i}} \cdot C_{liver,i}$$

- Liver

$$V_{liver,1} \cdot \dot{C_{liver,1}} = Q_H \cdot C_{rapid,1} + k_{a,1} \cdot C_{SI,1} - \frac{Q_H + CL_{bile,total,1} + CL_{CES,1} + CL_{3A4,1} + CL_{3A4,2}}{K_{P,H,1}} \cdot C_{liver,1}$$

$$V_{liver,2} \cdot \dot{C_{liver,2}} = Q_H \cdot C_{rapid,2} + k_{a,2} \cdot C_{SI,2} + \frac{CL_{CES,1}}{K_{P,H,1}} \cdot C_{liver,1} + \frac{CL_{CES,2}}{K_{P,H,4}} \cdot C_{liver,4} - \frac{Q_H + CL_{bile,total,2} + CL_{UGT}}{K_{P,H,2}} \cdot C_{liver,2}$$

$$V_{liver,3} \cdot \dot{C_{liver,3}} = Q_H \cdot C_{rapid,3} + k_{a,3} \cdot C_{SI,3} + \frac{CL_{UGT}}{K_{P,H,2}} \cdot C_{liver,2} - \frac{Q_H + CL_{bile,total,3}}{K_{P,H,3}} \cdot C_{liver,3}$$

$$V_{liver,4} \cdot \dot{C_{liver,4}} = Q_H \cdot C_{rapid,4} + k_{a,4} \cdot C_{SI,4} + \frac{CL_{3A4,2}}{K_{P,H,1}} \cdot C_{liver,1} - \frac{Q_H + CL_{bile,total,4} + CL_{CES,2}}{K_{P,H,4}} \cdot C_{liver,4}$$

$$V_{liver,5} \cdot \dot{C_{liver,5}} = Q_H \cdot C_{rapid,5} + k_{a,5} \cdot C_{SI,5} + \frac{CL_{3A4,1}}{K_{P,H,1}} \cdot C_{liver,1} - \frac{Q_H + CL_{bile,total,5}}{K_{P,H,5}} \cdot C_{liver,5}$$
